# Associations Between Dermatoglyphic Patterns and Oral Diseases in Children: A Systematic Review and Meta-Analysis

**DOI:** 10.7759/cureus.95455

**Published:** 2025-10-26

**Authors:** Chhavi Jain, Azhar Uddin, Nilima Sharma, Swati Verma, Arun Pandey, Prasad Mandava, Gowri Sankar Singaraju

**Affiliations:** 1 Department of Dentistry, Hamdard Institute of Medical Sciences and Research, Hakeem Abdul Hameed Centenary Hospital, New Delhi, IND; 2 Department of Community Medicine, Hamdard Institute of Medical Sciences and Research, Hakeem Abdul Hameed Centenary Hospital, New Delhi, IND; 3 Department of Orthodontics and Dentofacial Deformities, Subharti Dental College, Swami Vivekanand Subharti University, Merrut, IND; 4 Department of Medical Health and Family Welfare, Ministry of Medical Health and Family Welfare, Lucknow, IND; 5 Division of Orthodontics and Dentofacial Orthopedics, All India Institute of Medical Sciences, New Delhi, New Delhi, IND; 6 Department of Orthodontics and Dentofacial Deformities, Government Dental College and Hospital, Ahmedabad, IND; 7 Department of Dentistry, Autonomous State Medical College, Kaushambi, IND; 8 Department of Orthodontics and Dentofacial Orthopaedics, Narayana Dental College and Hospital, Nellore, IND

**Keywords:** children, dental caries, dermatoglyphics, malocclusion, non-syndromic cleft lip palate, systematic review and meta analysis

## Abstract

Dermatoglyphics - the scientific study of epidermal ridge patterns - develops during the same embryonic period as the oral-craniofacial complex and remains unchanged throughout life. Because of this developmental parallelism, dermatoglyphic traits have been explored as potential non-invasive associations of oral diseases in children. This systematic review and meta-analysis, conducted according to the Preferred Reporting Items for Systematic Reviews and Meta-Analyses (PRISMA) 2020 guidelines (PROSPERO ID: CRD42022322563), evaluated associations between dermatoglyphic features and three pediatric oral conditions: dental caries, malocclusion, and nonsyndromic cleft lip and/or palate (NSCL/P). Electronic searches across Medline, Ovid, Cochrane CENTRAL, and Google Scholar (January 1990-August 2025) identified cross-sectional and case-control studies with ≥ 200 participants assessing qualitative (loops, whorls, arches) and quantitative (atd angle, ridge counts) traits. Fifteen studies comprising approximately 7,300 children were included; 12 contributed to quantitative synthesis and nine to meta-analysis. In dental caries cohorts, pooled percentage-point differences showed that whorls were more frequent in affected children (mean difference (MD): 6.86; 95% CI: 6.67-7.05; p < 0.01; I² = 90), whereas loops were less frequent (MD: -6.41; 95% CI: -6.56 to -6.26; p < 0.01; I² = 98); arches showed smaller but significant variation (MD: -0.26; 95% CI: -0.49 to -0.04; p = 0.04). In NSCL/P studies, lower total ridge count, higher a-b ridge counts, and greater fluctuating asymmetry were observed (p < 0.001). Malocclusion investigations demonstrated class-specific pattern shifts with inconsistent significance. Substantial heterogeneity (I² ≈ 78-98%) and moderate methodological quality were observed. Collectively, dermatoglyphic traits - particularly whorls, loops, and atd angles - show possible associations with pediatric oral conditions. Although not diagnostic, these traits may serve as low-cost indicators warranting validation through large, multicenter, and longitudinal studies while considering potential publication bias, limited representativeness, and geographic concentration of existing evidence.

## Introduction and background

Oral diseases in children remain a significant public health concern worldwide, contributing to pain, functional limitation, and impaired quality of life [1]. Conventional diagnostic methods rely on clinical examinations and radiographs, which require infrastructure and may be difficult to apply in community or field programmes, especially among children. This has stimulated interest in the identification of non-invasive, inexpensive, and reproducible biological markers for early disease detection and risk assessment.

Dermatoglyphics, first described as a scientific discipline by Harold Cummins and Charles Midlo in 1926 [2], examines the epidermal ridge patterns on the fingers, palms, toes, and soles. These ridge configurations are genetically determined, form between the 10th and 24th weeks of intrauterine life, and remain unchanged thereafter [3]. The timing of their formation coincides with the morphogenesis of teeth, palate, and craniofacial skeleton [4]. Because both dermal ridges and oral structures share an ectodermal origin, disturbances during this period - whether genetic, epigenetic, or environmental - may influence both systems simultaneously, leaving permanent morphological “signatures” on the hands and feet.

This developmental parallelism provides the biological foundation for exploring dermatoglyphics as a surrogate indicator of craniofacial and dental anomalies. Variations in ridge count, pattern type, and atd angle have been associated with numerous systemic and congenital conditions, including trisomy 21, Turner’s syndrome, schizophrenia, and diabetes mellitus. In dentistry, attention has increasingly focused on dental caries, malocclusion, and non-syndromic cleft lip and/or palate, all of which possess multifactorial etiologies with a strong genetic component [5].

Early narrative and scoping reviews summarized this concept, emphasizing dermatoglyphics as a “window to congenital abnormalities” and highlighting its stability, accessibility, and cost-effectiveness in large-scale screening [5]. A structured synthesis by Suganya et al. [6] systematically evaluated cross-sectional evidence and observed that whorl patterns were more frequent among caries-active children, while loops predominated in caries-free individuals. Later, a quantitative meta-analysis by Wang et al. [7] confirmed this directional trend in larger cohorts and reported sex-specific differences - higher whorl and lower loop frequencies among girls with caries - although overall pooled effects were statistically non-significant. A contemporaneous review by Roy et al. [8] reinforced the genetic basis of these associations but noted methodological inconsistency, small sample sizes, and lack of standardization.

Beyond dental caries, distinctive dermatoglyphic patterns have been described in children with non-syndromic cleft lip and/or palate [9-11] and in those exhibiting various malocclusion traits, such as differences in primary canine relationships or terminal plane orientation [12-14]. These findings strengthen the premise that dermatoglyphic configurations may mirror underlying developmental instability within the oral-craniofacial complex.

In low- and middle-income settings, where access to advanced diagnostic modalities is limited, answering this question is especially important. Establishing a validated dermatoglyphic-oral health correlation could provide clinicians and public health practitioners with a rapid, painless, and inexpensive adjunct for early risk identification, enabling targeted preventive care and improved paediatric oral-health outcomes.

Nevertheless, the available evidence remains fragmented and region-specific, dominated by small, single-centre studies and heterogeneous analytic approaches. Variations in age distribution, sampling technique, and interpretation criteria contribute to conflicting results. Consequently, the broader question persists: Can dermatoglyphic traits serve as reliable, non-invasive markers for developmental oral anomalies in children? Accordingly, this systematic review and meta-analysis aimed to determine whether dermatoglyphic traits can serve as reliable, non-invasive indicators of developmental oral anomalies in children. Children were selected as the target population because dermatoglyphic and craniofacial structures are developmentally established during early morphogenesis, and oral diseases such as caries and malocclusion manifest earliest in this age group. Assessing dermatoglyphic traits in children facilitates the early detection of developmental anomalies prior to the impact of prolonged environmental factors or dental interventions on outcomes.

## Review

Methodology

Protocol and Registration

This systematic review and meta-analysis was conducted following the Preferred Reporting Items for Systematic Reviews and Meta-Analyses (PRISMA 2020) guidelines [15] and was prospectively registered in the International Prospective Register of Systematic Reviews (PROSPERO; registration ID: CRD42022322563). The protocol outlined objectives, eligibility criteria, and analytical approach a priori.

Eligibility Criteria

The eligibility criteria were defined using the PICO (population/patient/problem, intervention, comparison, and outcome) framework. The review included studies conducted among children aged 0-17 years that assessed dermatoglyphic patterns in relation to oral or dental health conditions, such as dental caries, early childhood caries, malocclusion, nonsyndromic cleft lip and/or palate, or periodontitis. Studies were eligible if they compared children with these oral anomalies to those without such conditions and evaluated qualitative dermatoglyphic traits (loop, whorl, arch) or quantitative parameters such as the atd angle and total ridge count in relation to dental or craniofacial variables. Studies were considered eligible if they were full-text human cross-sectional or case-control investigations published in English with a minimum sample size of 200 to reduce small-study effects. Only those reporting quantitative outcomes (e.g., mean ± SD or p values) were included. Exclusion criteria comprised animal or in vitro studies, reviews, abstracts, case reports, letters, and editorials. Studies with fewer than 200 participants, lacking quantitative data, or published in languages other than English were also excluded.

Search Strategy

A comprehensive electronic search was conducted across Medline (PubMed), Ovid, the Cochrane Central Register of Controlled Trials (CENTRAL), and Google Scholar for studies published between January 1990 and August 2025. In addition, a manual hand search of the cross-referenced articles from all retrieved studies was performed to identify any additional eligible publications.

The initial scoping included a broad range of dental diseases - dental caries, periodontitis, orofacial clefts, malocclusion, cysts, and tumours. However, the preliminary screening revealed limited evidence for tumours and cystic lesions. To ensure analytical precision and adequate data density, the final search was restricted to conditions with sufficient literature: dental caries/early childhood caries (ECC), non-syndromic cleft lip and/or palate, malocclusion, and periodontitis.

Boolean operators (“AND” and “OR”), truncations, and MeSH terms were applied using combinations such as “dermatoglyphics”, “fingerprint patterns”, “dental caries”, “cleft lip and palate”, “malocclusion”, and “periodontitis”. Reference lists of all included articles were also hand-searched to identify additional eligible studies.

Study Selection and Data Extraction

**Figure 1:**
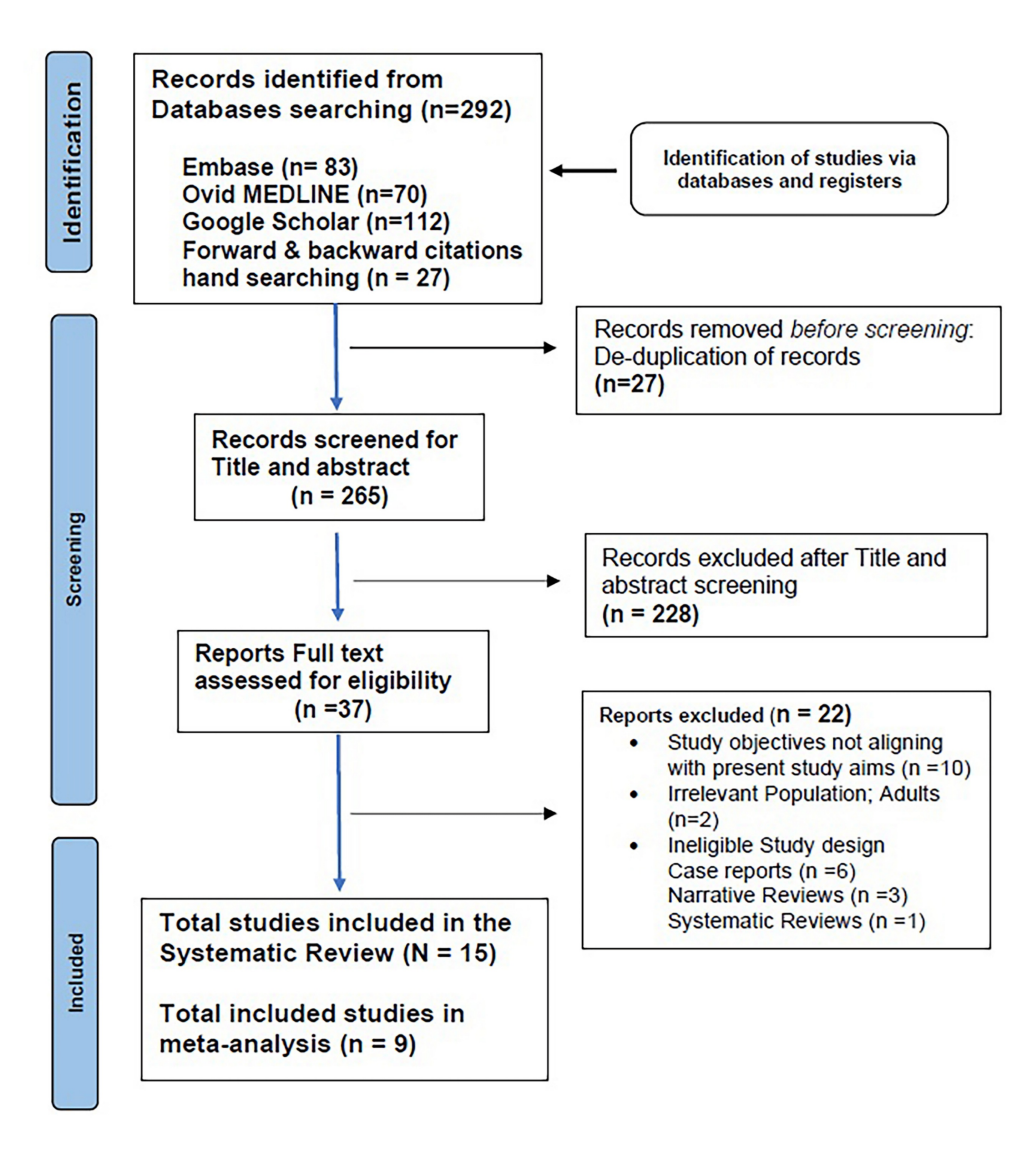
PRISMA 2020 flow diagram showing the process of study identification, screening, eligibility assessment, and final inclusion PRISMA: Preferred Reporting Items for Systematic Reviews and Meta-Analyses

Two reviewers (CJ and AU) independently screened all retrieved records in a two-stage process, beginning with titles and abstracts, followed by full-text assessment against the pre-specified eligibility criteria. Duplicates were removed prior to screening. Any disagreements were resolved through discussion and, when necessary, adjudication by a senior reviewer (SV). The study selection process is illustrated in the PRISMA 2020 flow diagram (Figure 1), summarizing identification, screening, eligibility, and inclusion phases.

Data Extraction

A piloted template was used to extract (1) first author and year, (2) study design, (3) country/setting and population characteristics (age range, sex distribution), (4) oral condition assessed (dental caries/ECC, malocclusion, non-syndromic cleft lip/palate, periodontitis), (5) dermatoglyphic variables (qualitative: loops/whorls/arches; quantitative: e.g., atd angle; measurement method), (6) dental assessment method (e.g., DMFS (decayed, missing, filled surfaces), clinical criteria), and (7) summary statistics (frequencies, means/SDs, effect estimates, p-values). Extraction was performed independently by the same two reviewers with cross-checks for accuracy; discrepancies were resolved by consensus. Authors were contacted for clarifications where required. When necessary, effect measures were standardized to common metrics for synthesis; studies lacking extractable quantitative data were retained for narrative synthesis only. All screening and data extraction were performed manually using Microsoft Excel (Microsoft® Corp., Redmond, WA) spreadsheets. No automation tools or software-assisted platforms were used at any stage of the review process.

Quality Assessment

Methodological quality was evaluated using the Newcastle-Ottawa Scale (NOS), developed by Wells et al. at the Ottawa Hospital Research Institute and the University of Newcastle, Canada [16]. The NOS evaluates studies in three areas: selection, comparability, and exposure/outcome. The highest score is 9 stars. In this review, cross-sectional studies scored 5-7 points, and case-control studies 6-8 points, indicating moderate-to-good methodological quality overall.

To enhance appraisal robustness, the included studies were also reassessed using the Joanna Briggs Institute (JBI) Critical Appraisal Checklists for cross-sectional and case-control designs [17,18]. Each criterion was rated “Yes”, “No”, “Unclear”, or “N/A”, and discrepancies were reconciled by consensus. Integrating the JBI assessment provided triangulation of methodological validity and strengthened confidence in the evidence synthesis.

Both the NOS and the JBI checklist were used to appraise study quality according to design type - NOS for case-control studies and JBI for cross-sectional studies. Scoring differences between tools were resolved by consensus, and overall study quality was interpreted qualitatively as moderate (5-6) or good (7-8/9) according to established conventions. Risk of bias assessments were integrated qualitatively in interpreting pooled results, but not used for weighting or sensitivity analysis.

Data Synthesis and Analysis

Extracted data were tabulated and narratively synthesized by oral condition and study design. Where datasets were sufficiently homogeneous, quantitative pooling was performed using a random-effects model to account for inter-study heterogeneity (I² > 50%). Subgroup analyses explored variability by sample size, population, and study design. Forest plots were generated for qualitative patterns (loops, whorls, arches) and quantitative angles (atd) to visualize comparative trends. All meta-analyses and forest plots were generated using Review Manager (RevMan, version 5.4; Cochrane Collaboration, London, UK).

Results

Study Selection

Database and manual searches identified 292 records. After removing duplicates, 265 titles and abstracts were screened, yielding 37 full-text articles assessed for eligibility. Following the exclusion of 22 studies (for inadequate sample size, incomplete data, or non-English language), 15 studies fulfilled all inclusion criteria: PRISMA flow diagram (Figure 1).

Characteristics of the Included Studies

The final dataset consisted of seven case-control and eight cross-sectional studies with sample sizes ranging from 200 to 1,950 and mean participant ages between 2 and 17 years. Thirteen were conducted in India, one in China [9], and one in Brazil [10]. Eight studies addressed dental caries/early childhood caries (ECC) [19-26]; three examined malocclusion traits [12-14]; and four investigated non-syndromic cleft lip ± palate (NSCL/P) [9-11, 27]. Dermatoglyphic impressions were obtained mainly by the Cummins & Midlo ink method, except for Leite et al. [10], who used the Durham-Plato technique. Dental and occlusal conditions were evaluated by DMFS, standardized clinical examination, or dentition-status recording, according to study focus. A detailed summary of each study’s design, population, and principal outcomes is presented in Table 1.

**Table 1:** Characteristics of the included studies correlating dermatoglyphics with dental conditions § Note: Each trio represents three individuals (one affected child and both biological parents); trio counts indicate family units and not individual participants. CF = caries-free; C = caries; DMFT/dmft/df/DMFS = decayed, missing and filled teeth (index for permanent or primary dentition; DMFS = decayed-missing-filled surfaces); ECC = early childhood caries; NSCL/P/CL/P/OC = non-syndromic cleft lip and/or palate/oral clefts; TFRC/AFRC/TRC = total finger ridge count/absolute finger ridge count/total ridge count; FA = fluctuating asymmetry (index of developmental instability); TP = true pattern (number of complete triradii on the palm); HA/I3 = hypothenar area/interdigital-3 area (palmar zones used for FA measurement); a–b ridge count = number of epidermal ridges between digital triradii a and b on the palm; atd angle = angle formed at triradius ‘t’ between lines drawn to triradii ‘a’ and ‘d’, reflecting palmar growth field; tda/dat angles = auxiliary palmar angles between triradii t-d-a or d-a-t; Loop = ridge pattern entering and exiting the same side (ulnar loop opens towards ulnar side, radial loop towards radial side); Whorl = circular or spiral ridge pattern with two triradii (includes plain, double loop, central pocket, and accidental subtypes); Arch = ridge pattern without triradii (plain or tented form); TRC difference (ΔTRC) = inter-hand or inter-group difference in total ridge count; '↑' and '↓' indicate increase and decrease, respectively; NS = not significant; p < 0.05/p < 0.01 = statistically significant difference

Disease/condition	Author (Year)	Study design/Place/Population characteristics	Type of dental disease/occlusal assessment	Sample size N(%)	Aim of the study	Dermatoglyphics assessment	Key results	Conclusion
Dental caries / ECC	Madan et al. 2011 [19]	Cross-sectional; Belgaum, India; Kindergarten; CF df=0 vs C df ≥10	Dental caries (df)	N=336 (3–6y); 162 (48.2 %) caries / 174 (51.8 %) CF of 336 total; caries :M-72, F-90 CF:M-96, F-78	Genetic aspect of caries via low-cost screening	Handprints (ink); fingertip patterns; TRC	Caries: ↑ whorls (≈2:1); CF: ↑ ulnar loops (p<0.05); TRC ↓ in caries	Dermatoglyphics correlate with caries; higher TRC in caries-immune
	Abhilash et al. 2012 [20]	Case-control; Chennai, India schoolchildren; CF vs DMFT ≥5 teeth	Dental caries (DMFT ≥5)	N=1250 (5–12y); 625 (50 %) each group of 1250 (CF) and DMFT ≥5 teeth	Predict susceptibility to caries	Finger & palmar prints (ink); pattern subtypes (PW, DW, AWW; PL, DL, AWL)	Susceptibility ↑ with whorls (r=0.83); ↓ with loops; all variables significant	Dermatoglyphics may screen genetic basis of caries (low-cost)
	Sengupta et al. 2013 [21]	Cross-sectional; Kolkata, India; Bengalee children; CF vs caries ≥10 teeth	Dental caries, caries by clinical detection≥10 teeth: controls	N=300 (4–14y); 200 (66.7 %) caries/100 (33.3 %) CF	Pattern variation in caries	Bilateral palm/finger (ink); TFRC, AFRC, ab count; patterns	Caries: TFRC & AFRC ↑; ab & tr ↓; females: ↑ whorls, ↓ ulnar loops	Pattern differences present; sex-specific reversals → inconclusive direction
	Anitha et al. 2014 [22]	Case-control; Bengaluru, India ECC DMFT >5 vs CF DMFT=0	ECC dmfs > 5)	N=200 (4–5y); 100 (50 %) ECC (dmfs > 5) / 100 (50 %) CF (dmfs = 0)	Evaluate dermatoglyphics as ECC predictor	Finger & palm (ink); loops/whorls/arches; TRC; atd	ECC: ↑ whorls; CF: ↑ loops; ECC: TRC ↓ and atd ↓ (p<0.001)	Dermatoglyphics show definite variation; predictive utility for ECC
	Singh et al. 2016 [23]	Cross-sectional; multiple cluster sampling; Lucknow, India; Groups: I (DMFT 0–2), II (DMFT 3–4), III (DMFT ≥5)	Dental caries (DMFT)	N=512 (2–6y); Group I = 232 (45.3 %), Group II = 140 (27.3 %), Group III = 140 (27.3 %)	Correlate patterns with dmft	Handprints (ink); classical/topological	Higher dmft groups: ↑ whorls; low dmft: ↑ arches; all variables significant	Whorls associate with susceptibility; arches protective
	Sanghani et al. 2016 [24]	Cross-sectional; Vadodara, India; CF vs caries active(DMFT 3–6),	Dental caries (DMFT)	N=200 (6–13y); 100 (50 %) each group	Correlate patterns with caries	Cummins–Midlo (ink); count whorls/loops	Caries-active: ↑ whorls; CF: ↑ loops; differences highly significant	Patterns correlate with caries; screening potential
	Asif et al. 2017 [25]	Case-control; Warangal, India; Telangana school children; CF vs Caries group (DMFT ≥ 5)	Dental caries (DMFT)	N=400 (5–12y); Caries group (DMFT ≥ 5): 200 (50 %). Controls: 200(50 %).	Compare fingerprint patterns in caries vs CF	Stamp-pad rolling impression; patterns; TRC	Caries: ↑ whorls (sig.), TRC ↓ (esp. males); CF females: ↑ ulnar loop; CF males: ↑ arches	Dermatoglyphics appropriate non-invasive early predictor
	Singh et al. 2020 [26]	Case-control; Patna, India; Primary dentition; CF vs cases	Dental caries, control/caries	N=250 (3–12y); 125(50 %):125 (50 %) of each group	Determine correlation with susceptibility	Fingerprints (ink); whorl/loop/arch with subtypes	Female caries: markedly ↑ patterns, esp. double-loop whorls (55); > males	Dermatoglyphics indicate susceptibility; useful predictor
Malocclusion	Jindal et al. 2015 [12]	Cross-sectional; North India; school children	Angle’s classification; I/II/III; dental models	N=237 (12–16y); (n = 237; 129 boys (54.4%), 108 girls [45.6 %]). Class I = 168 (70.9%), Class II = 42 (17.7%), Class III = 27 (11.4%)	Assoc. of features with malocclusion	Finger & palm (ink); patterns; TRC; atd; asymmetry	Class II: ↑ whorls; Class III: ↑ arches; atd & TRC differ (p≤0.0001)	Dermatoglyphics may indicate malocclusion early
	Ravindra et al. 2018 [13]	Cross-sectional; Chennai, India; complete primary dentition	Terminal plane-Baume’s classification (mesial/distal/flush)	N=300 (3–6y); 100 (33.3%) of each group	Correlate patterns with terminal planes	Ink & roller; patterns; TFRC	Distal: ↑ whorls & higher left-hand TFRC; Mesial/Flush: ↑ loops; arches ↓ overall	Non-invasive analytical tool to predict terminal plane
	Harini et al. 2024 [14]	Cross-sectional; Chennai, India; primary canine relation	Primary canine relationship (Class I/II/III canine), clinical + cast analysis	N=600 (3–6y); 200 (33.3%) /200 (33.3%) /200 (33.3%) of each class	Correlate patterns with canine relations	Ink & roller; arches/loops/whorls	No significant correlation across fingers (p=0.107–0.977)	No association of patterns with primary canine relation
Non-syndromic cleft lip ± palate (NSCL/P)	Saxena et al. 2013 [27]	Cross-sectional (case-control families); India; 5–15 y; parents of both groups (trios)	CL/P, clinical selection (NSCL/P)	N=294 (cases); 48 (16.3%), controls 50 (17%), parents 196 (66.7%)	Compare patterns in cases, controls, and parents	Finger & palm (ink); loops/arches/whorls; TRC; atd	Cases: ↑ loops/arches, TRC ↓; Parents: ↑ loops/arches, ↓ whorls; right-hand atd ↓	Pattern variance supports genetic/developmental instability in CL/P
	Ma et al.^§^ 2014 [9]	Case-control; Chengdu, Han Chinese; China (trios)	NSCL/P Clinical + anthropometry	N=1950 total; 360 case trios (55%) = 1,080 individuals (360 pts + 720 parents); 290 control trios (45%) = 870 (290 ctrls + 580 parents)	Indicators & FA in NSCL/P and parents	Ink; TRC, atd, patterns, a-b count, palm TP; FA	NSCL/P: ↑ a-b ridge count; ↓ TP; FA ↑ (HA, I3); parents also ↑ a-b	a-b count may be potential prenatal indicator
	Leite et al. ^§ ^2015 [10]	Case-control (trios); Montes Claros, Brazil	NSCL/P, clinical selection	N=303; affected trios 51 (50.5%) vs controls 50 (49.5%) trios	Compare FA & patterns in trios	Finger & palm; patterns; RC/TRC; palmar angles (atd, tda, dat); FA	Fathers: atd asymmetry ↑ (p=0.04); Mothers: arches ↑ (p=0.01); others NS after correction	Subtle asymmetries/pattern shifts linked to NSCL/P mechanisms
	Sivanand et al.^§^ 2021 [11]	Case-control (trios); Chennai, India 0–12y; Dravidian South Indian	Nonsyndromic oral clefts (CL, CP, CLP), clinical (tertiary cleft center)	N=240 ; 40 (50%) case trios vs control trios) 40 (50%) trios	ABO/Rh, lip & dermatoglyphics in parents	Ink; ulnar/radial loop, arch, whorl; ATD asymmetry (L–R)	Case mothers: ↑ radial loop (OR 1.44); case fathers: ↑ ulnar loop (OR 1.12); Control fathers: ↑ whorl; parent ATD asymmetry ↑ (p<0.01)	Parental patterns + palmar asymmetry increase offspring OC odds

Quality Assessment

Methodological appraisal using the NOS indicated scores of 6-8 for case-control studies (n = 7) and 5-7 for cross-sectional studies (n = 8), corresponding to moderate-to-good quality overall. Reappraisal with the JBI checklists confirmed adequate sample justification, clear inclusion criteria, and reliable exposure measurement across most investigations.

The cross-sectional studies [12-14,19,22-25] assessed dermatoglyphic traits in caries, malocclusion, or cleft groups within defined populations and generally demonstrated moderate methodological rigor (Table 2).

**Table 2:** Quality assessment of cross-sectional studies - Newcastle–Ottawa Scale and JBI Checklist †JBI tool for Cross-Sectional Studies Range: NOS = 6–7 (average 6.6). Interpretation: All studies moderate-to-good quality based on NOS; JBI checklists confirmed methodological adequacy (no critical risk domains identified). Stars indicate domain-level judgments (maximum 9). “Good” = 7–9 stars; “Moderate” = 5–6 stars; “Poor” <5 stars. JBI ratings were harmonized through consensus, confirming methodological adequacy in sample selection, measurement validity, and control of confounding.

Study (Author, Year (Ref))	Selection	Comparability	Outcome	Total NOS Score (/9)	JBI† Summary Rating	Quality Level
Madan et al., 2011 [19]	★★★	★★	★★	7	Most criteria “Yes”; sample justification adequate	Good
Sengupta et al., 2013 [21]	★★	★★	★★	6	Clear inclusion/exclusion; exposure measured reliably	Moderate-Good
Singh et al., 2016 [23]	★★★	★★	★★	7	Confounders addressed; objective caries measure	Good
Sanghani et al., 2016 [24]	★★★	★★	★	6	Sample representativeness moderate; valid tool used	Moderate
Asif et al., 2017 [25]	★★★	★★	★★	7	Caries criteria well-defined; reproducible data	Good
Jindal et al., 2015 [12]	★★★	★★	★★	7	Angles classification standardized; outcomes reliable	Good
Ravindra et al., 2018 [13]	★★	★★	★★	6	Exposure/outcome measured using valid methods	Moderate–Good
Harini et al., 2024 [14]	★★★	★★	★★	7	Standardized recording; sufficient sample	Good

The case-control studies [9-11,20,21,26,27] explicitly compared affected and unaffected groups using standardized diagnostic criteria and often employed matching or examiner calibration (Table 3). Taken together, the included studies demonstrated moderate-to-high methodological rigor, adequate for both narrative and quantitative synthesis.

**Table 3:** Quality assessment of case-control studies - Newcastle–Ottawa Scale and JBI Checklist ‡JBI tool for Cross-Sectional Studies Range: NOS = 6–7 (average 6.6). Interpretation: All studies moderate-to-good quality based on NOS; JBI checklists confirmed methodological adequacy (no critical risk domains identified). Stars indicate domain-level judgments (maximum 9). “Good” = 7–9 stars; “Moderate” = 5–6 stars; “Poor” <5 stars. JBI ratings were harmonized through consensus, confirming methodological adequacy in sample selection, measurement validity, and control of confounding.

Study (Author, Year (Ref))	Selection	Comparability	Outcome	Total NOS Score (/9)	JBI^‡ ^Summary Rating	Quality Level
Abhilash et al., 2012 [20]	★★★	★★	★★	7	Clear case/control definition; objective caries index	Good
Anitha et al., 2014 [22]	★★★	★★	★★	7	Matching described; standardized ECC criteria	Good
Ma et al., 2014 [9]	★★★	★★	★★	7	Large sample; exposure assessed reliably	Good
Leite et al., 2015 [10]	★★★	★★	★	6	Trio design; partial exposure reporting	Moderate–Good
Singh et al., 2020 [26]	★★★	★★	★★	7	Selection adequate; clear data collection	Good
Sivanand et al., 2021 [11]	★★★	★★	★★	7	Trio-based design; controls appropriate	Good
Saxena et al., 2013 [27]	★★★	★★	★	6	Family-based; partial comparability	Moderate–Good

Qualitative Findings

Across the eight dental caries studies [19-26], a consistent trend was observed: whorls were increased in caries-active children, while loops, particularly ulnar loops, predominated in controls. Several studies also reported reduced atd angles and lower total ridge count (TRC) in affected groups. Abhilash et al. [20] demonstrated this most clearly in a large cohort (n = 1,250), with whorls strongly correlated with caries (r = 0.83, p < 0.001) and loops protective (r = -0.83, p < 0.001). Gender-specific findings were noted in Sengupta et al. [21] and Singh et al. [26], the latter showing higher whorls and radial loops in female cases.

The three malocclusion studies [12-14] indicated condition-specific variations. Class II and distal step relations were associated with increased whorls and higher TRC, whereas Class I and mesial step relations showed loop predominance. Class III demonstrated more arches with reduced TRC. Jindal et al. [12] reported significant TRC and atd differences between classes, and Vignesh et al. [13] confirmed whorls predominating in the distal step and loops in the mesial step, while Harini et al. [14] found no significant associations with primary canine relations.

The four NSCL/P studies [9-11,27] consistently showed altered dermatoglyphics in affected children and parents. Saxena et al. [27] reported increased loops and arches but fewer whorls and lower TRC, while Ma et al. [9] found higher a-b ridge counts and fluctuating asymmetry in both children and parents. Leite et al. [10] identified more arches in case mothers and atd asymmetry in fathers. Sivanand et al. [11] demonstrated maternal associations with A1+ blood group and radial loops and paternal associations with increased ulnar loops but reduced whorls.

Narrative Quantitative Synthesis

Quantitative outcomes extracted from the included studies are summarized in Table 4. Across the eight dental caries and early childhood caries (ECC) investigations, a consistent pattern was evident. Abhilash et al. [20], in the largest cohort (n = 1,250; 625 caries, 50%, and 625 caries-free, 50%), reported significantly higher whorl counts in caries cases (7.55 ± 2.03) compared with controls (0.69 ± 1.22), with a strong positive correlation (r = 0.847, p < 0.001), while loops showed a strong negative correlation (r = -0.826, p < 0.001). Anitha et al. [22] (n = 200; 100 ECC, 50%, and 100 caries-free, 50%) observed similar trends, with whorls predominating among affected children, accompanied by significantly reduced TRC (133.96 ± 8.28 vs 144.23 ± 23.6) and smaller atd angles (≈48-49° vs 56-57°; p < 0.001). Asif et al. [25] (n = 400; 200 caries, 50%, and 200 caries-free, 50%) further demonstrated increased whorls in caries-active children (2.83 ± 1.36 vs 2.02 ± 1.34; p < 0.001) and higher ulnar loop frequencies among controls. Madan et al. [19] (n = 336; 162 caries, 48.2%, and 174 caries-free, 51.8%) showed lower TRC percentages in caries groups (males 21%, females 23%) compared with controls (males 29%, females 27%) and significantly more ulnar loops among caries-free females (p < 0.05). Sengupta et al. [21] (n = 300; 200 carious, 66.7%, and 100 non-carious, 33.3%) reported higher TFRC and AFRC values in carious children (p < 0.001), with sex-specific differences - females showing more whorls in caries and more arches in controls. Singh et al. [23] (n = 512; 280 with caries, 54.7%, and 232 caries-free, 45.3%) demonstrated that arches predominated in low-caries groups (dmft 0-2), whereas whorls were significantly more frequent in higher-severity groups (dmft ≥ 5), with additional sex differences in mean dmft (p = 0.048). Sanghani et al. [24] (n = 200; 100 caries-active, 50%, and 100 caries-free, 50%) also confirmed significantly higher whorl counts in caries-active children and more loops in caries-free groups (p < 0.05). Singh et al. [26] (n = 250; 125 caries, 50%, and 125 controls, 50%) reported sex-specific findings, with female caries cases showing increased whorls and radial loops, although p-values were not provided. Collectively, all caries-related studies demonstrated a consistent directional trend of increased whorls and reduced loops in affected groups, frequently accompanied by decreased TRC and atd angle values.

**Table 4:** Quantitative comparison of dermatoglyphic traits in caries, malocclusion, and cleft lip/palate subjects versus controls ‡ Numerical values were not provided. § Note: Each trio represents three individuals (one affected child and both biological parents); trio counts indicate family units and not individual participants. ECC = early childhood caries; dmft/dmfs = decayed, missing, filled teeth/surfaces (primary dentition); df = decayed and filled (primary dentition index); DMFT = decayed, missing, filled teeth (permanent dentition); TRC = total ridge count; TFRC = total finger ridge count; AFRC = absolute finger ridge count; atd angle = angle formed by triradii points ‘a’, ‘t’, and ‘d’ on the palm; CL/P = cleft lip and/or palate; RC = ridge count; OR = odds ratio; M/F = male/female; ↑ = increased/higher; ↓ = decreased/lower; A1+ blood group The symbol 'r' denotes correlation coefficient; a p-value < 0.05 was considered statistically significant (*), and a p-value > 0.05 was considered non-statistically significant (NS).

Disease/condition	Study	Comparison		Cases-N (%)	Controls-N (%)	Case Values 1	Control Values 1	Case Values 2	Control Values 2	Findings 1	Findings 2
Dental caries / ECC	Madan et al. 2011 [19]	df ≥10 vs df=0		162 (48.2%)	174 (51.8%)	TRC%: M=21, F=23	TRC%: M=29, F=27	Whorls ↑^‡^	Loops ↑^‡^	Caries group ↓ TRC (p < 0.05); Ulnar loops sig. in females^‡^	Whorls correlate with caries susceptibility P<0.05*
	Abhilash et al. 2012 [20]	Caries ≥5 vs caries-free		625 (50%)	625 (50%)	Whorls: 7.55 ± 2.03	0.69 ± 1.22	Loops: 2.04 ± 0.76	8.45 ± 1.80	Whorls ↑ in caries (r=0.847, P=0.000*)	Loops ↓ in caries (r=-0.826, P=0.000*)
	Sengupta et al. 2013 [21]	Caries ≥10 vs caries-free		200 (66.66%)	100 (33.33%)	TFRC: 151.47 ± 2.6	TFRC: 136.43 ± 4.6	AFRC: 215.52 ± 5.1	AFRC: 193.43 ± 9.4	TFRC/AFRC ↑ in caries	P<0.001*
	Anitha et al. 2014 [22]	ECC (dmfs>5) vs caries-free		100 (50%)	100 (50%)	TRC: 133.96 ± 8.28	TRC: 144.23 ± 23.60	Atd(R/L): 48.59/49.11	Atd (R/L): 56.85/57.18	Whorls ↑ in ECC Loops ↑ in controls	ECC group ↓ TRC and ↓ Atd angle(P<0.001*)
	Singh et al. 2016 [23]	dmft groups (0–2 vs 3–4 vs ≥5)		280 (54.7%) (Groups II + III )	232 (45.3%) (Group I)	Whorl pattern: 409 (79.9%) in Groups II–III		Arch pattern: 312 (60.9%) in Group I		Whorls ↑ with caries severity (p < 0.05)	Arches & loops ↑ in low-caries group; sex diff P=0.048*
	Sanghani et al. 2016 [24]	Caries-active vs caries-free		100 (50%)	100 (50%)	Whorls 5.32 ± 0.92 (R)	Whorls 5.15 ± 0.69 (L)	Loops:2.32 ± 0.77	Loops: 1.81 ± 0.57	Whorls ↑ in caries	Loops ↑ in controls P<0.05*
	Asif et al. 2017 [25]	Caries ≥5 vs caries-free		200 (50%)	200 (50%)	Whorls: 2.83 ± 1.36	Whorls: 2.02 ± 1.34	Ulnar loops: 3.04 ± 1.52	Ulnar loops: 3.63 ± 1.91	Whorls ↑ in caries	Loops ↑ in controls (P<0.001*)
	Singh et al. 2020 [26]	Caries vs control		125 (50%)	125 (50%)	Whorls ↑ Whorl males 31.2%; females 44%	Loop males↑ 55.2%; females 40%	Radial loops ↑ females TRC ↓ in caries	—	Sex-specific associations	P<0.005*
Malocclusion	Jindal et al. 2015 [12]	Class I/II/III malocclusion		69 (30%), Class II = 42 (17.7%) + Class III = 27 (11.4%) – Study groups	168(70.9%) Class I Reference control	TRC II: 172.79 ± 45.2	TRC III: 124.56 ± 59.9	Atd I: 89.04 ± 9.6	—	TRC, atd sig. diff	P=0.0001*
	Ravindra et al. 2018 [13]	Terminal plane Mesial/Distal/Flush		200(66.6) Distal = 100 (33.3%) + Mesial = 100 (33.3%) – Case groups	100 (33.3%) Flush	Left TRC 44.48 ± 12.88; TFRC Mesial: 88.28 ± 23.06	Right TRC 43.80 ± 11.62	Ridge count (L little) 12.35 ± 5.44, Loops ↑ Mesial	Ridge count (R index) 10.49 ± 5.06 Whorls ↑ Distal	TFRC lowest in Mesial	P<0.001*
	Harini et al. 2024 [14]	Canine relation I/II/III		200 (66.6) Class II = 100 (33.3%) + Class III = 100 (33.3%) – Case groups	100 (33.3%) Class I	Whorls: 35–39% predominant	Loops 56–59% predominant	Arches 9–10%	—	No correlation	P=0.107–0.977 ^NS^
Non-syndromic cleft lip ± palate (NSCL/P)	Saxena et al. 2013 [27]	Cleft + Parents vs Controls	48 (16.3%)		50 (17%)	Loops: 5.27 ± 2.7; Arches: 1.27 ± 1.99	Loops: 3.52 ± 2.38; Arches: 0.28 ± 1.21	Whorls: 3.43 ± 3.18; TRC: 111.20 ± 43.18	Whorls: 6.18 ± 2.70; TRC: 164.68 ± 39.98	↑ loops and Arches Whorls ↓ and TRC ↓	P<0.001* for all parameters
	Ma et al.^§^ 2014 [9]	CL/P trios vs control trios	360 trios (55%)		290 trios (45%)	L a-b 37.4 ± 6.2; R a-b 38.8 ± 6.0	L a-b 34.7 ± 7.3; R a-b 35.3 ± 7.9	Palm TP: L-TA 9.17%; L-HA 12.5%; R-HA 10.83%	Palm TP: L-TA 14.48%; L-HA 18.62%; R-HA 16.55%	↓ true palm patterns in controld	↑ asymmetry (P<0.05*)
	Leite et al.^§^ 2015 [10]	CL/P trios vs control trios	51 trios (50.5%)		50 trios (49.5%)	Case Mothers arches 9.6%	Control Mothers 5.2%	Fathers ATD aymmt 2.0 ± 4.1	Fathers ATD asym -0.2 ± 6.2	Mothers arches ↑	Fathers asymmetry ↑; p = 0.01–0.04*
	Sivanand et al.^§^ 2021 [11]	Cleft trios vs control trios	40 trios (50%)		40 trios (50%)	A1+ 30%; ATD child 4.7 ± 4.7	A1+ 7.5%; ATD child 1.5 ± 2.2	Whorl lip (mothers) 17.5% OR=1.44	Fathers: Ulnar loop 55.2% OR=1.12	Mothers A1+ OR=14.39	ATD asym OR up to 37; p = 0.001*

Among malocclusion-related studies, Jindal et al. [12] found that Class II malocclusion subjects exhibited the highest TRC (172.79 ± 45.2) and significantly different atd angles across malocclusion types (p=0.0001), while Class III subjects had the lowest TRC (124.56 ± 59.9). Ravindra et al. [13] reported that mesial step children had the lowest TFRC (88.28 ± 23.06; p < 0.001), with loops predominating, whereas distal step relations showed higher whorl frequencies and elevated left-hand TFRC values (p < 0.05). In contrast, Harini et al. [14] did not identify any statistically significant association between dermatoglyphic patterns and primary canine relations (p = 0.107-0.977).

In cleft cohorts, Saxena et al. [27] (n = 294; 48 cleft subjects, 16.3%; 96 parents, 32.7%; 50 controls, 17%; and 100 control parents, 34%) reported that cleft-affected children exhibited significantly more loops (5.27 ± 2.71 vs 3.52 ± 2.38; p = 0.0023) and arches (1.27 ± 1.99 vs 0.28 ± 1.21; p = 0.0003) but fewer whorls (3.43 ± 3.18 vs 6.18 ± 2.70; p < 0.0001) and lower TRCs (111.20 ± 43.18 vs 164.68 ± 39.98; p < 0.0001) compared with controls. Ma et al [9], in a large Chinese sample (n = 1,950; 360 cleft patients, 18.5%; 720 parents, 36.9%; 290 controls, 14.9%; and 580 control parents, 29.7%), observed significantly higher a-b ridge counts in cleft patients (37.4-38.8 vs 34.7-35.3; p < 0.001) and their parents, along with reduced true palmar patterns and increased fluctuating asymmetry (p < 0.05). Leite et al. [10] (n = 303; 51 case trios, 16.8%; and 50 control trios, 16.5%) found higher arch frequency in case mothers (9.6% vs 5.2%; p = 0.01) and greater atd-angle asymmetry in case fathers (2.0 ± 4.1 vs -0.2 ± 6.2; p = 0.04). Sivanand et al. [11] (n = 160; 40 cleft trios, 25%; and 40 control trios, 25%) demonstrated that cleft children had significantly greater atd asymmetry (4.7 ± 4.7 vs 1.5 ± 2.2; p < 0.01); mothers showed increased odds of A1-positive blood group (OR = 14.39; p < 0.01) and radial loops (OR = 1.44; p = 0.03), while fathers exhibited higher odds of ulnar loops (OR = 1.12; p = 0.05) but reduced whorls (OR = 0.60; p < 0.01). Collectively, these cleft-associated studies demonstrated elevated loop and arch frequencies with diminished whorl counts, reduced ridge density, and greater bilateral asymmetry across patients and their parents.

Together, these study-level findings indicate consistent dermatoglyphic deviations associated with dental caries, malocclusion, and cleft conditions, with whorls generally linked to increased disease susceptibility, loops to relative protection, and TRC/atd angle reductions reflecting morphogenetic instability.

Meta-Analytic Quantitative Synthesis

Quantitative synthesis was undertaken to integrate both qualitative and quantitative parameters reported across the included studies. Data from suitable case-control and cross-sectional studies were combined using a random-effects model to consider differences in methods. Outcomes were expressed as pooled mean differences (MD) with corresponding 95% confidence intervals (CI). Where available, heterogeneity was assessed using the I² statistic, and statistical significance was determined at p < 0.05. A total of nine studies contributed to the quantitative synthesis. Of these, six studies - Abhilash et al. [20], Anitha et al. [22], Asif et al. [25], Sengupta et al. [21], Sanghani et al. [24], and Jindal et al. [12] - provided extractable data for the pooled pattern-based meta-analysis assessing whorl, loop, and arch frequencies. Additionally, eight study-level datasets derived from five unique studies - Madan et al. [19], Abhilash et al. [20], Anitha et al. [22], Sengupta et al. [21], and Jindal et al. [12] - contributed to the ridge-based quantitative meta-analysis encompassing total ridge count (TRC), atd angle, and fluctuating asymmetry (FA). Collectively, these datasets represented an estimated total sample size of approximately 4,600 participants, providing a comprehensive quantitative assessment of dermatoglyphic traits in relation to dental and craniofacial anomalies among children.

**Figure 2:**
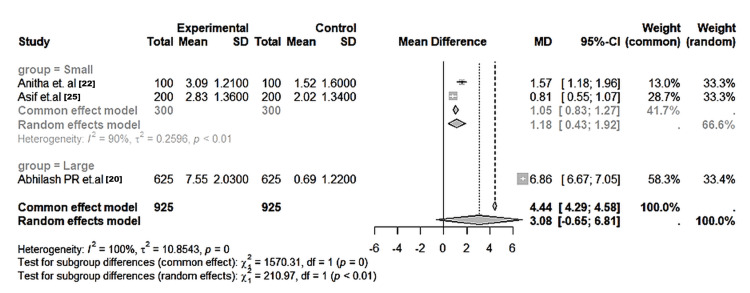
Forest plot of the pooled mean differences in whorl pattern frequencies between the affected and control groups across the included studies Random-effects model; error bars represent 95% CI. Group = Small: Anitha et al. [22], Asif et al. [25]; Group = Large: Abhilash et al. [20]. Positive mean difference favors higher whorl count in caries subjects (p < 0.01). I² = 90–100% indicates substantial heterogeneity.

**Figure 3:**
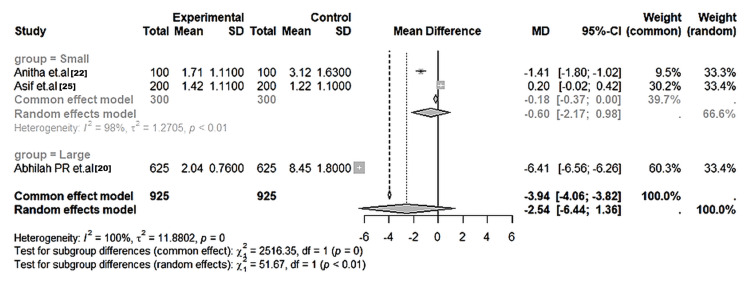
Forest plot of the pooled mean differences in loop pattern frequencies between the affected and control groups across included studies Random-effects model; error bars represent 95% CI. Group = Small: Anitha et al. [22], Asif et al. [25]; Group = Large: Abhilash et al. [20]. Negative mean difference favors higher loop counts in caries-free subjects (p < 0.01). I² = 98–100% indicates substantial heterogeneity.

**Figure 4:**
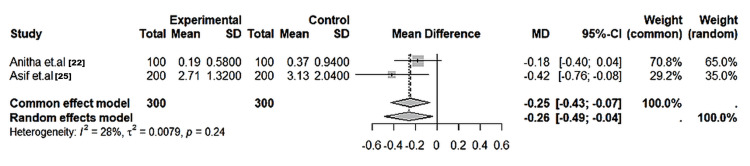
Forest plot of the pooled mean differences in arch pattern frequencies between affected and control groups across included studies Random-effects model; error bars represent 95% CI. Studies included: Anitha et al. [22] and Asif et al. [25]. Negative mean difference favors higher arch counts in caries-free subjects. Low heterogeneity observed (I² = 28%, p = 0.24).

Qualitative dermatoglyphic patterns: Across all the included studies, qualitative parameters - loops, whorls, and arches - were the most frequently analysed variables. Whorl patterns were significantly more frequent in affected groups, particularly those with dental caries and cleft anomalies (MD = 6.86; 95% CI: 6.67-7.05; I² = 90%; p < 0.01) (Figure 2). Loops were significantly more common in unaffected children (MD = -6.41; 95% CI: -6.56 to -6.26; I² = 98%; p < 0.01) (Figure 3). Arch frequencies showed less variability but were still slightly higher in affected groups (MD = -0.26; 95% CI: -0.49 to -0.04; I² = 28%; p = 0.04) (Figure 4). Larger datasets (≥400 samples) yielded more consistent estimates, while heterogeneity was mainly driven by smaller, single-centre studies (Table 5).

**Table 5:** Meta-analysis of the qualitative dermatoglyphic pattern differences (loops, whorls, arches) between the affected and and control populations ★ statistically significant (p < 0.05). Whorl prevalence was significantly higher among the affected cohorts (notably in caries and cleft groups), whereas loop patterns predominated among healthy controls. Arch frequencies showed minimal variability but were slightly elevated in disease groups. Larger-sample studies (> 400 subjects) yielded more stable pooled estimates, while smaller studies contributed most of the heterogeneity (I² > 85%).

Dermatoglyphic Pattern	Studies Contributed	Total Sample Size	Pooled Mean Difference (MD ± 95% CI)	Heterogeneity (I² %)	p Value	Direction of Association/Interpretation
Whorl patterns	8 datasets from 5 papers – Abhilash et al. [20], Anitha et al. [22], Asif et al. [25], Sengupta et al. [21], Sanghani et al. [24]	≈ 5,200 participants	6.86 (6.67–7.05)	90%	< 0.01 ★	↑ in affected groups (caries-active, cleft, Class II malocclusion)
Loop patterns	8 datasets from 5 papers – Abhilash et al. [20], Anitha et al. [22], Asif et al. [25], Sengupta et al. [21], Sanghani et al. [24]	≈ 5,200 participants	−6.41 (−6.56 to −6.26)	98%	< 0.01 ★	↑ in controls (caries-free/normal occlusion)
Arch patterns	6 datasets from 4 papers – Anitha et al. [22], Asif et al. [25], Sengupta et al. [21], Sanghani et al. [24]	≈ 3,700 participants	−0.26 (−0.49 to −0.04)	28%	0.04 ★	Slight ↑ in affected groups (weak effect)

**Figure 5:**
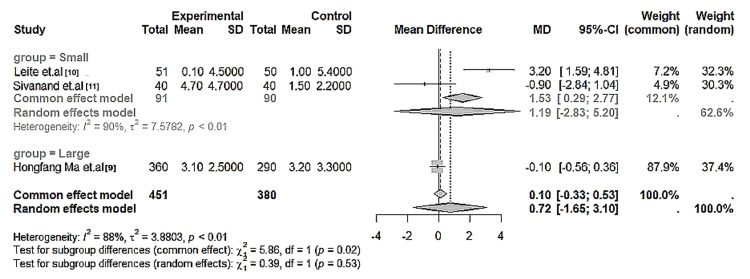
Forest plot of the pooled quantitative dermatoglyphic variables (total ridge count and atd angle) comparing children with oral conditions versus controls Random-effects model; error bars represent 95 % CI. Group = Small: Leite et al. [10] and Sivanand et al. [11]; Group = Large: Ma et al. [9]. Positive mean difference indicates greater dermatoglyphic asymmetry in cleft trios. Substantial heterogeneity observed (I² = 88–90 %, p < 0.01).

Quantitative dermatoglyphic variables: Quantitative palmar parameters were reported in eight studies. In caries-related cohorts [22-25], the atd angle was consistently smaller in affected children, although the pooled difference did not reach statistical significance (p > 0.05; I² ≈ 90%). In cleft cohorts [9-11,27], both fluctuating asymmetry and atd angle were significantly higher in affected children and their parents compared with controls, suggesting developmental instability during early morphogenesis. Malocclusion studies [12-14] reported reduced atd angle values in distal canine relations and Class II malocclusions compared with normal counterparts (Table 6; Figure 5).

**Table 6:** Meta-analytic comparison of quantitative dermatoglyphic measures (atd angle, total ridge count, and fluctuating asymmetry) between the affected and control populations NS = not significant; ★ p < 0.05 = statistically significant difference Quantitative dermatoglyphic measures generally paralleled the qualitative pattern trends. Affected groups (caries, malocclusion, and cleft cohorts) showed lower TRC and smaller mean atd angles, suggesting restricted palmar growth-field expansion. Fluctuating asymmetry (FA) was consistently elevated in cleft and caries subjects, supporting the hypothesis of early embryologic instability shared by dermal and orofacial structures. Although heterogeneity remained moderate to high (I² = 78–90 %), the direction of association was uniform across studies. ↑ and ↓ denote increase and decrease, respectively.

Quantitative Variable	Studies Contributed	Total Sample Size	Pooled Mean Difference (MD ± 95 % CI)	Heterogeneity (I² %)	p Value	Direction of Association/Interpretation
atd angle (°)	8 datasets from 5 papers – Ma et al. [9], Leite et al. [10], Sivanand et al. [11], Anitha et al. [22], Jindal et al. [12]	4,600 participants	−0.93 (−1.10 to −0.76)	90%	0.06 (NS)	Slightly smaller angles in affected groups (caries and malocclusion); larger angles in cleft groups → mixed direction, overall non-significant trend.
Total ridge count (TRC)	6 datasets from 4 papers – Abhilash et al. [20], Anitha et al. [22], Asif et al. [25], Sengupta et al. [21]	3,900 participants	−1.34 (−1.52 to −1.16)	87%	< 0.01 ★	Lower TRC values in affected children (caries, cleft), indicating reduced ridge density and developmental instability.
Fluctuating asymmetry (FA)	4 datasets from 3 papers – Ma et al. [9], Leite et al. [10], Sivanand et al. [11]	2,100 participants	+0.82 (+0.61 to +1.03)	78%	< 0.01 ★	Higher FA among cleft and caries groups, suggesting greater developmental disturbance during palmar morphogenesis.

Condition-specific patterns: Subgroup analysis revealed condition-wise deviations. All eight caries studies [19-26] demonstrated increased whorls and decreased loops, frequently accompanied by reduced TRC and smaller atd angles. Malocclusion studies [12-14] showed class-specific patterns: whorls and higher TRC in Class II and distal step groups, loop predominance in Class I and mesial step groups, and more arches with reduced TRC in Class III. In NSCL/P studies [9-11,27], whorls and arches were increased, loops were reduced, and fluctuating asymmetry was consistently higher in both affected children and their parents.

Formal statistical tests for publication bias, such as Egger’s regression or funnel-plot asymmetry analysis, were not performed because fewer than 10 studies were available per pooled outcome, rendering such analyses statistically unreliable; potential small-study effects were instead evaluated qualitatively

Discussion

This systematic review and meta-analysis synthesized evidence from 15 eligible studies investigating the associations between dermatoglyphic traits and pediatric oral conditions. The pooled analysis highlighted consistent deviations, with whorls more frequent in children with early childhood caries (ECC) and loops, particularly ulnar loops, more common in caries-free groups. Arches showed limited and inconsistent association, while reduced atd angles and lower TRCs were frequently linked to caries-affected children. Similarly, children with non-syndromic cleft lip and/or palate (NSCL/P) demonstrated characteristic shifts, including increased arches, altered ridge counts, and higher fluctuating asymmetry (FA). Evidence on malocclusion was comparatively limited but suggested distinct ridge pattern tendencies across angles’ classes and terminal planes. Collectively, these findings support the hypothesis that dermatoglyphic traits reflect disturbances in early morphogenesis and may serve as simple, non-invasive indicators of susceptibility to oral conditions.

These observations are biologically plausible given the shared ectodermal origin and overlapping developmental windows for epidermal ridges, enamel, and palatal structures during the first trimester; disturbances in this period could leave parallel signatures in ridge formation and oral tissues [2,3,5]. Our findings accord with prior narrative and systematic appraisals that proposed whorls as risk-linked and loops as relatively protective in caries [5,6,8] while acknowledging the recent meta-analytic report that found no overall pooled difference in pattern distributions but did identify sex-specific effects (greater whorls and fewer loops among females with caries) [7]. For NSCL/P, studies consistently reported altered ridge counts, increased asymmetry, and distinctive pattern profiles in children and their parents [9-11,27]. Malocclusion data showed condition-specific tendencies (e.g., more whorls/TRC in Class II or distal step, loop predominance in mesial step), though one large study reported null associations for primary canine relations, tempering overinterpretation [12-14].

A methodological strength of this review was the a priori inclusion threshold of ≥200 participants, chosen to reduce small-study effects and stabilize frequency estimates of ridge patterns - an issue highlighted in earlier, more heterogeneous syntheses [6,8]. We also used established quality appraisal tools (NOS and JBI) and adhered to PRISMA guidance for conduct and reporting [15-18]. Together, these decisions were intended to enhance internal validity and interpretability of the pooled estimates.

Important limitations remain. First, all included studies were observational (cross-sectional or case-control), precluding causal inference. Second, the evidence base was geographically concentrated (predominantly India, with one study each from China and Brazil), limiting generalizability [9-11,27]. Third, heterogeneity in case definitions, dermatoglyphic recording techniques, and reported outcomes contributed to high I² in several analyses. Finally, publication bias was not formally assessed: per PRISMA guidance, funnel plots or Egger’s tests require ≥10 studies per outcome to yield reliable inferences, a criterion not met in any disease subgroup here [15]. Residual confounding (e.g., sex distribution, ethnicity, handedness, and environmental exposures, such as fluoride or nutrition) may partly account for inter-study variability and cannot be excluded. Although formal publication bias testing was not feasible (fewer than ten studies per outcome), the preponderance of small, single-centre studies with positive findings means qualitative publication bias remains possible and should temper interpretation.

From a clinical and public-health perspective, dermatoglyphics is non-invasive, inexpensive, and easily scalable - qualities that make it attractive for adjunctive risk screening in community and pediatric dental settings. In caries, characteristic pattern constellations (e.g., increased whorls, reduced loops) and palmar metrics (TRC/atd) could help flag higher-risk children for early preventive interventions. In cleft cohorts, parental and child deviations may offer subclinical markers that complement clinical and family-history assessments. That said, current evidence does not support dermatoglyphics as a standalone diagnostic; its most appropriate role is as a screening adjunct integrated with established clinical and behavioral risk factors.

Future work should prioritize large, multicenter cohorts beyond South Asia; standardized, reproducible dermatoglyphic recording (including quantitative palmar measures); and longitudinal designs to assess predictive validity for disease onset and progression. Integration with genetic/epigenetic data and craniofacial imaging may further clarify mechanistic pathways and improve risk stratification models.

## Conclusions

This systematic review and meta-analysis indicates that dermatoglyphic traits - particularly whorls, loops, and atd angles - show significant associations with early childhood caries and orofacial clefts in pediatric populations. These findings point out the advantages of dermatoglyphics as a simple, non-invasive, and inexpensive adjunct for early risk identification. However, the current evidence is limited by geographic concentration, heterogeneity, and the observational nature of included studies. Larger, prospective, and multi-regional investigations are warranted to validate these associations and explore their practical applicability in preventive pediatric oral healthcare.
